# Novel exosome-associated LncRNA model predicts colorectal cancer prognosis and drug response

**DOI:** 10.1186/s41065-025-00445-0

**Published:** 2025-05-16

**Authors:** Chi Zhou, Qian Qiu, Xinyu Liu, Tiantian Zhang, Leilei Liang, Yihang Yuan, Yufo Chen, Weijie Sun

**Affiliations:** 1 Department of General Surgery, The First Affiliated Hospital of Bengbu Medical University, Bengbu, China; 2 Department of Pathology, The First Affiliated Hospital of Anhui Medical University, Hefei, China; 3 Bengbu Medical University, Bengbu, China; 4 Department of Medical Oncology, First Affiliated Hospital of Bengbu Medical University, Bengbu, China; 5 Department of Gynecologic Oncology, Zhejiang Cancer Hospital, Hangzhou, China; 6 Department of General Surgery, Nanjing Drum Tower Hospital, Affiliated Hospital of Medical School Nanjing University, Nanjing, China

**Keywords:** Colorectal cancer, LncRNA, EALncRI, Integrating analysis, Immune, Prognosis, MIR4713HG, Oncogene

## Abstract

**Background:**

Exosomes are extracellular vesicles that carry various biological substances and have potential as functional mediators in cancers. However, little is known about special molecules in colorectal cancer (CRC) exosomes and their immunological functions.

**Aims:**

Using genomic data from the TCGA-CRC cohort, we constructed a prognostic model based on exosome-related lncRNA for the first time, and the biological role of MIR4713HG in CRC was deeply analyzed.

**Method:**

In this study, we downloaded the gene expression data and clinical data of CRC from the TCGA database. The limma package, SVM-REF and univariate Cox analysis were used to screen out core ERG (CERG) in CRC. LASSO and multivariate Cox regression analyses were used to filter out CERG-related LncRNA and construct a risk score. We explored the distribution and expression levels of ERG in immune cell types by scRNA-seq data. xCell was used to calculate the infiltration levels of stromal cells and immune cells in CRC. KM plotter was used for immunotherapy evaluation of core ERG. Next, we further provide colony formation assay, Transwell assay and xenograft models to understand the carcinogenic effect of MIR4713HG.

**Result:**

First, 43 differentially expressed ERG and 7 CERG were obtained. We explored the expression and distribution levels of CERG in 9 types of cells by scRNA-seq data. In addition, two key exosome-associated LncRNA (MIR4713HG and ZEB1-AS1) were obtained, and a risk score (EALncRI) was constructed. EALncRI could accurately predict the prognosis of CRC. Based on the EALncRI, we constructed a nomogram that is easy to use in clinical practice, which can more accurately and stably predict the prognosis of CRC patients. Furthermore, EALncRI was significantly correlated with the expression of 5 HLA molecules and 13 immune checkpoint molecules. MIR4713HG showed a good predictive effect in the overall survival of patients with immunotherapy evaluation. Knocking down the expression of MIR4713HG significantly inhibited proliferation and migration, and also impaired subcutaneous tumor growth in nude mice.

**Conclusion:**

In this study, a variety of machine learning algorithms were used to construct the EALncRI based on ERG, which can effectively predict the prognosis and distinguish the immune landscape of CRC. More importantly, we conducted an in-depth study on MIR4713HG, which may become an important therapeutic target in CRC.

**Supplementary Information:**

The online version contains supplementary material available at 10.1186/s41065-025-00445-0.

## Introduction

Colorectal cancer (CRC) has approximately 1.93 million new cases and 0.90 million deaths annually, making it one of the most common and deadliest malignant tumors worldwide [1]. Due to the disease’s insidious onset and the lack of sensitive biomarkers, many patients are diagnosed at an advanced stage, with a 5-year survival rate of only about 10% [2]. Moreover, new cases and deaths from CRC will further increase until 2040 [3]. Therefore, there is a clinical need to find new prognostic markers and treatment targets for CRC patients.

Exosomes are a class of extracellular vesicles with an average diameter of approximately 100 nm that are secreted by eukaryotic cells and widely distributed in body fluids (blood, urine, saliva, etc.) [4]. Studies have shown that the biological functions of exosomes depend on their loaded cargo, including proteins, lipids and nucleic acids [5–7]. Due to this property, exosomes have an inherent advantage as noninvasive novel markers. In addition, exosomes can regulate tumor formation, metastasis, angiogenesis, drug resistance, etc [7]. Therefore, a systematic study of exosome-associated genes in CRC will help to understand the pathogenesis of CRC.

Long noncoding RNA (lncRNA) usually refers to a class of RNA that cannot encode proteins with a length of more than 200 nt [8]. LncRNA can regulate genes both transcriptionally and posttranscriptionally to exert biological functions [9]. Dysregulation of lncRNA expression is ubiquitous in various cancers and significantly regulates cancer initiation and progression [10]. For example, lncRNA FOXD2-AS1 regulates OSCC progression by regulating the MiR-185-5p pathway [11]. lncRNA can selectively act as “cargo” loaded by exosomes, facilitating intercellular communication [12]. In addition, exosome-derived lncRNA can regulate tumor development and have potential as diagnostic and prognostic markers for cancer [12, 13]. Currently, many studies have explored the role of gene-lncRNA network analysis in tumors [14–17]. However, there is currently no exosome-associated lncRNA model for accurately predicting the prognosis and immune status of CRC.

In this research, a variety of machine learning algorithms were used to construct the lncRNA risk index (EALncRI) based on exosome-related genes (ERGs), which can effectively predict the prognosis of CRC and distinguish the immune landscape. In addition, we conducted an in-depth study on MIR4713HG, which has not yet been explored in the model, and showed that knocking down the expression of MIR4713HG can significantly weaken the proliferation, migration and invasion abilities of CRC cells and the ability of tumor growth in vivo.

## Methods and materials

### Data sources

We downloaded clinical prognosis data and gene expression data (FPKM) of CRC (including rectal and colon cancer cohort) from the TCGA database. In addition, 120 ERGs were downloaded from the exoRBase database (http://www.exorbase.org/) and ExoBCD database (https://exobcd.liumwei.org/), see Supplementary Table 1 for details. Samples with incomplete data were excluded from the clinical correlation analysis. Prognostic analysis excluded samples with missing survival data and zero survival time. Single-cell RNA sequencing (scRNA-seq) data of CRC comes from GSE16655 (https://www.ncbi.nlm.nih.gov/geo/query/acc.cgi?acc=GSE16655).

### scRNA-seq preprocessing

Based on our previous research [18], we used the Seurat package to preprocess the scRNA sequencing data of CRC patients. Briefly, the content of mitochondrial genes in cells is evaluated using the PercentageFeatureSet function. In this study, we analysed cells with less than 5% mitochondrial genes and more than 1500 genes. The resolution is set to 0.25, and the principal components are set to 20. Cell clustering was performed based on the 1500 genes with the greatest differential expression between cells.

### ERG-associated LncRNA screening

We first perform a difference analysis on ERG, and then further filter it through the machine learning algorithm SVM-RFE and define it as SR-ERG. Next, we used univariate Cox (uni-cox) regression analysis to perform prognostic analysis on SR-ERG, screened out SR-ERG with prognostic significance, and then defined them as core ERG (CERG). Finally, we obtained CERG-associated LncRNA by coexpression analysis.

### Construction of the EALncRI

To avoid overfitting, we sequentially screened CERG-related LncRNA by uni-cox, LASSO and multivariate Cox (mul-cox) regression analysis, and constructed a risk score based on the results of mul-cox regression analysis. The formula is as follows: EALncRI = expression of MIR4713HG * coefficient of MIR4713HG + expression of ZEB1-AS1 * coefficient of ZEB1-AS1.

### Nomogram construction

We integrated the EALncRI and common clinicopathological parameters to construct a new nomogram and evaluated the stability and accuracy of the nomogram through the c-index and ROC curve.

### GO and KEGG analysis

First, we analysed the differentially expressed genes (DEGs) between the low EALncRI group and the high EALncRI group and then used “clusterProfiler”, “org.Hs.eg.db”, “enrichplot”, and “ggplot2” to perform GO and KEGG analysis on the DEGs to explore the biological processes and signaling pathways enriched by differential genes.

### Assessment of the tumor microenvironment and drug sensitivity analysis

First, we calculated the infiltration levels of 64 types of stromal and immune cells in CRC patients using xCell [19] and analysed the correlation between EALncRI and the infiltration levels of 64 types cells. Second, we calculated the somatic mutations of CRC patients by the maftools package and analysed the top ten most mutated genes in the low EALncRI group and the high EALncRI group. Then, we analysed the correlation of EALncRI with 24 HLA molecules and 48 immune checkpoint molecules (ICM). Finally, IC50 values for our two common CRC chemotherapy drugs were predicted using the oncopredict package [20] and evaluated the difference in the sensitivity of patients to chemotherapeutic drugs in the low EALncRI group and the high EALncRI group.

### Cell culture and lentiviral transfection

The CRC cell (HT29, SW480 and HCT116) was purchased from ATCC Cell Bank in the United States. HT29, SW480 and HCT116 was cultured in DMEM medium containing 1% penicillin-streptomycin and 10% fetal bovine serum in an incubator at 37 °C and 5% CO2. SH-NC and SHMIR4713HG lentiviruses were purchased from Guangzhou RIBOBIO Co., Ltd. The detailed sequences are as follows, sh-NC: 5’-TTCTCCGAACGTGTCACGT-3’; sh-MIR4713HG-1: 5’-TTCCTTGATATGATGTTCAAACC-3’; sh-MIR4713HG-2: 5’-TAGTTTTTGGACTCTTGAAGATG-3’. The lentiviral transfection steps were carried out according to our previous research report [21].

### RNA extraction, qRT‒PCR, colony formation assay, and transwell assay

The detailed steps of RNA extraction, qRT‒PCR, colony formation assay, Transwell assay, and animal experiment were performed as described in our previous research [21–23]. The sequences of primers are detailed in Supplementary Table 2.

### Xenograft tumor animal experiments

Male nude mice aged 4–6 weeks were purchased from Gempharmatech Co., Ltd. The experimental group cells (HT29, 5 × 10^6) were implanted subcutaneously into the nude mice. The size of the subcutaneous tumor (long diameter and short diameter) was observed and measured every week. After four weeks, the mice were euthanized, the subcutaneous tumor was removed, and the tumor weight was recorded.

### Statistical analysis

All bioinformatics analyses were performed in R language software (R: a language and environment for statistical computing; version 4.2.3; https://www.R-project.org/). Correlation analysis was performed by the Spearman method. The Wilcoxon test was used to analyse the difference between the two groups. Survival curves are drawn using the Kaplan-Meier (KM) method, and differences are calculated using the log-rank test. *p* < 0.05 indicates that the difference is statistically significant.

## Result

### Screening of CERG-related LncRNA

**Fig. 1:**
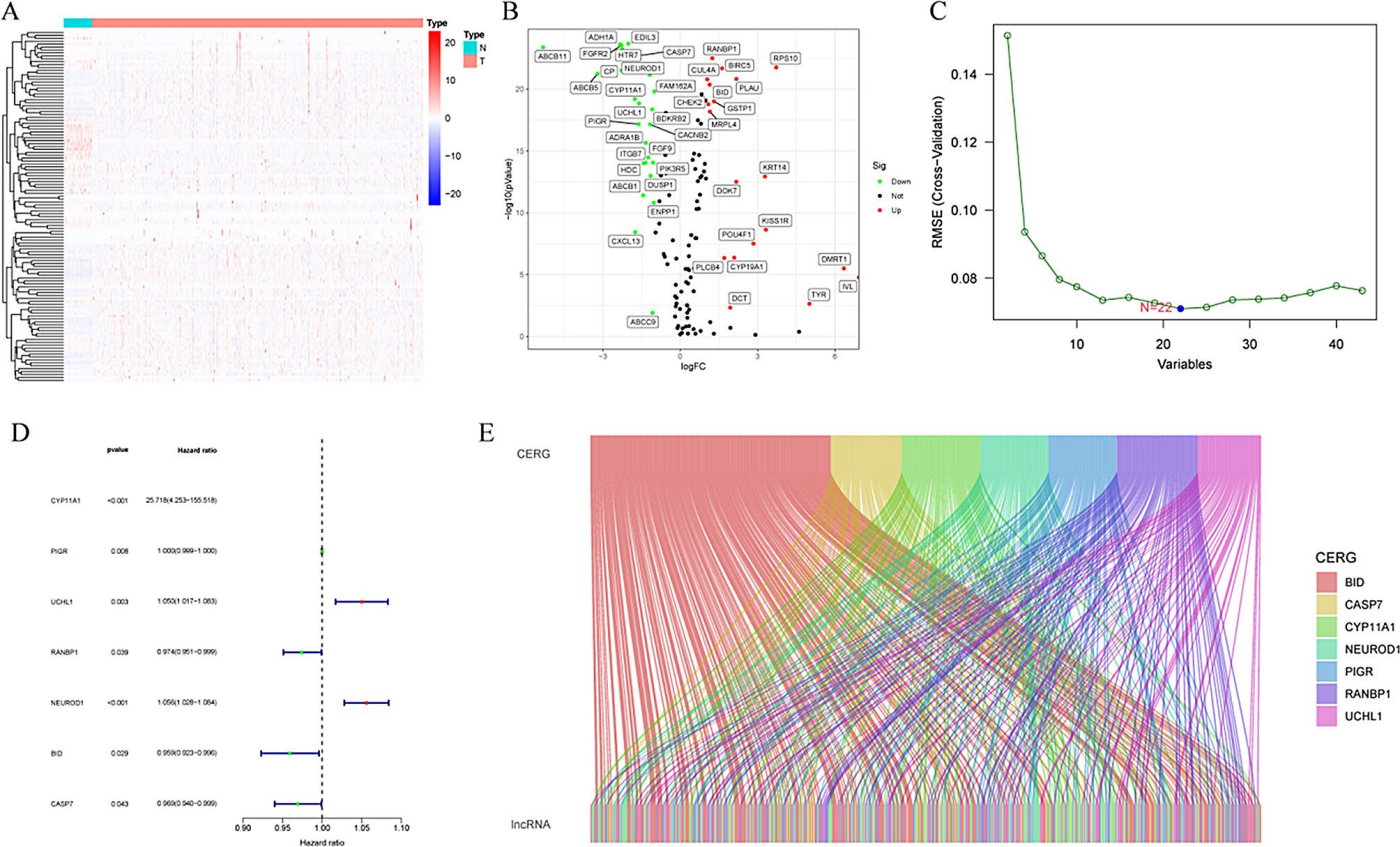
CERG-related lncRNA screening. (**A**) Heatmap of ERG expression in the TCGA cohort. (**B**) Volcano plot for ERG differential analysis. (**C**) SVM-RFE analysis for differentially expressed ERGs. (**D**) Univariate Cox regression analysis of SR-DEGs. (**E**) Coexpression network diagram of CERG and lncRNA

First, we conducted differential analysis on 120 ERGs, including 43 DEGs, including 18 DEGs with up-regulated expression and 25 DEGs with down-regulated expression. These results were visualized using heatmaps and volcano maps (Fig. 1A-B). Then, we used the machine learning algorithm SVM-RFE to further screen these 43 DEGs and finally obtained 22 DEGs and defined them as SR-DEGs (Fig. 1C). The 22 SR-DEGs were then subjected to prognostic analysis using univariate Cox regression analysis, and 7 core ERGs (CERGs) were finally obtained (Fig. 1D). Finally, exosome-associated LncRNA were obtained by coexpression analysis (Fig. 1E).

### Expression profiles of CERG in different cell subpopulations

**Fig. 2:**
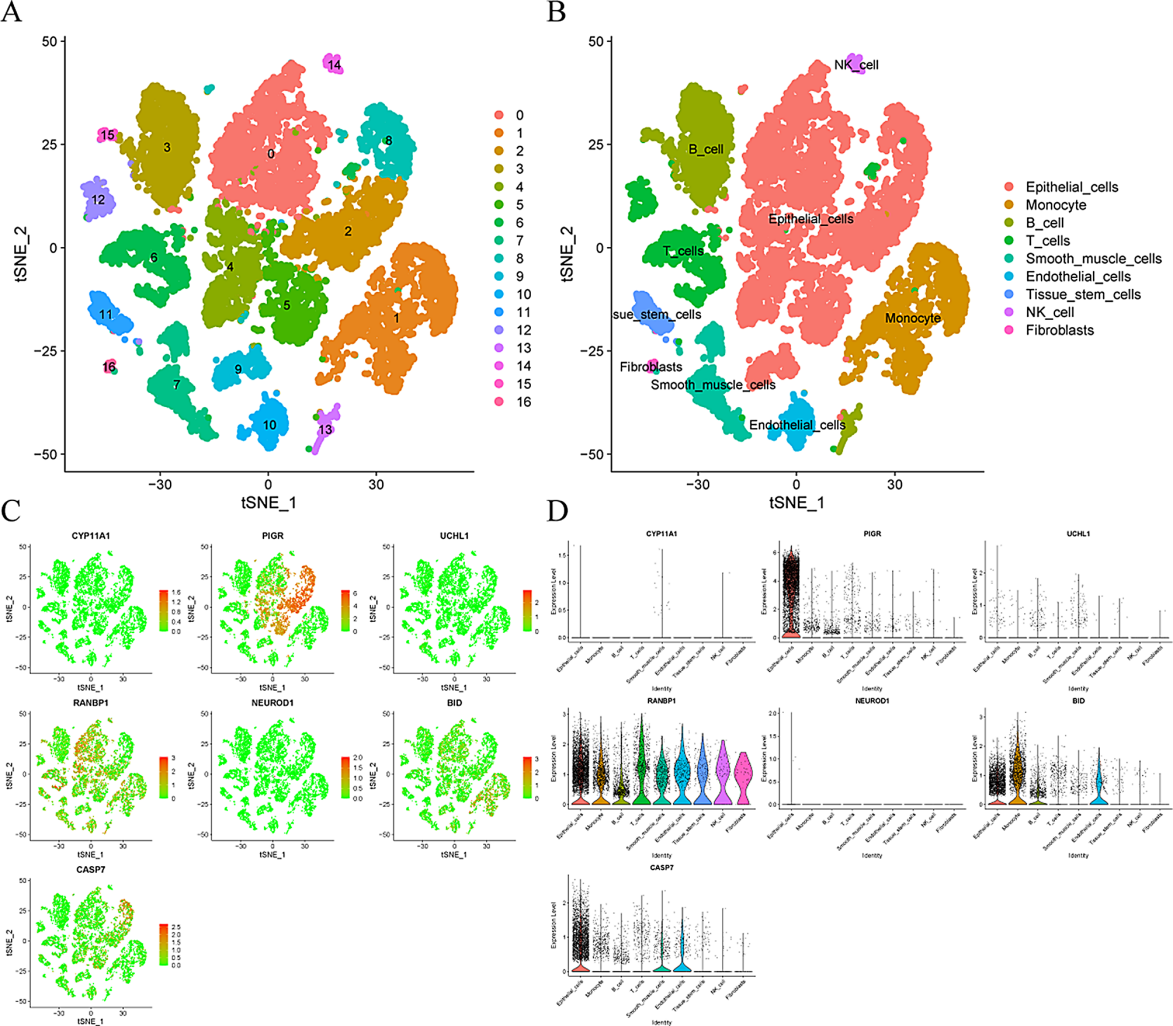
scRNA-seq analysis of CRC samples. (**A**) tSNE analysis for cell cluster classification. (**B**) Annotated by the “SingleR” package for 9 cell types. (**C**-**D**) Expression distribution and level of CERG in 9 cell types

Cells of the CRC samples were sorted into 16 cell clusters by tSNE analysis (Fig. 2A). Then, the 16 cell clusters were further classified into 9 cell types by “SingleR” package [24] (Fig. 2B), including epithelial cells, T cells, B cells, monocytes, endothelial cells, smooth muscle cells, tissue stem cells, NK cells, and fibroblasts. On this basis, we explored the distribution and expression levels of CERG (Fig. 2C–D). Briefly, CYP11A1, UCHL1, and NEUROD1 were expressed at low levels in each cell type, whereas RANBP1 was expressed at a high level in all cell types.

### Construction and verification of EALncRI

**Fig. 3:**
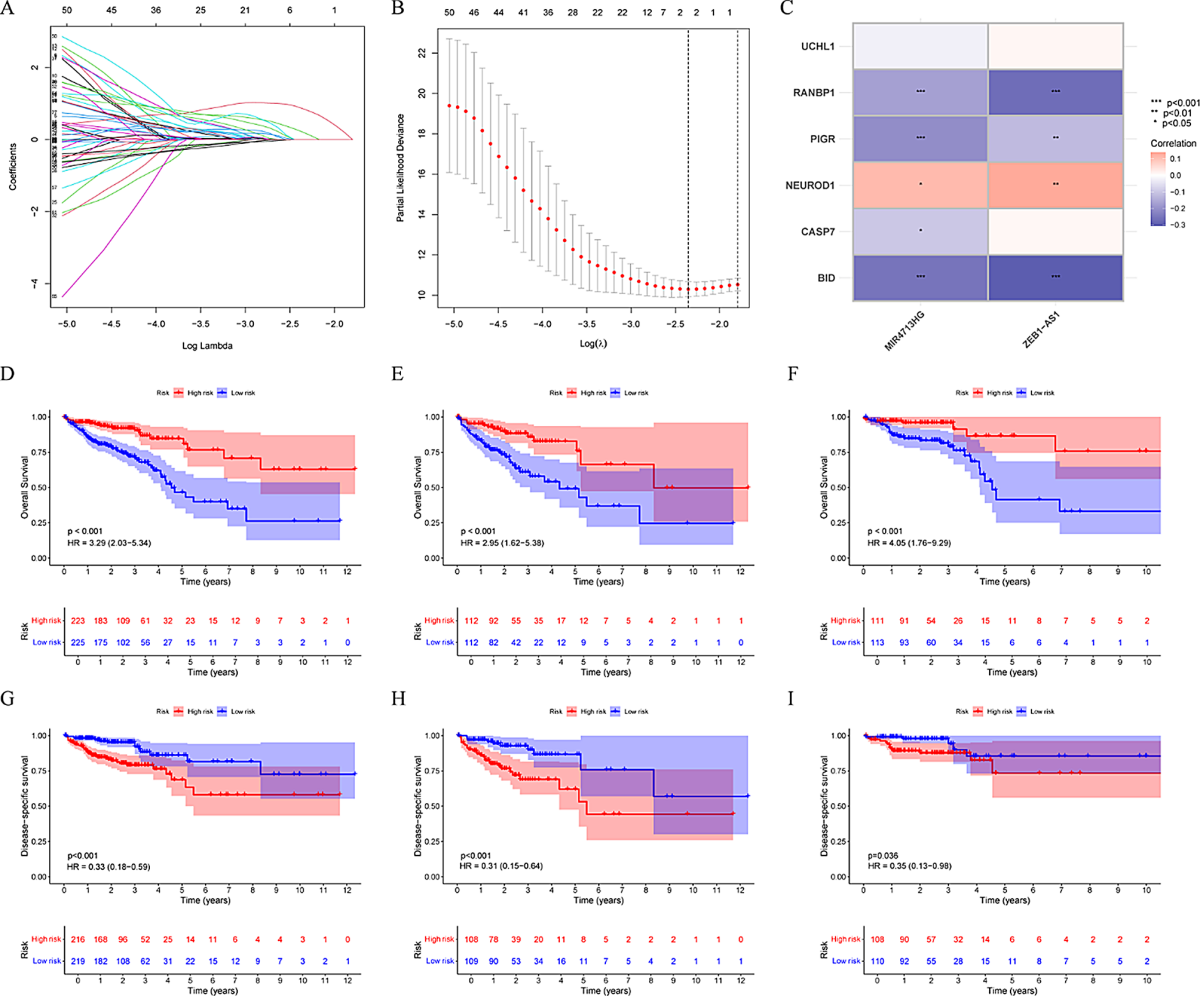
Construction and verification of EALncRI. (**A**-**B**) LASSO analysis of prognosis-related EALncRI. (**C**) Correlation heatmap of MIR4713HG and ZEB1-AS1 with CERG. (**D**-**F**) KM curves of OS for the total cohort, development cohort and verification cohort. (**G**-**I**) KM curves of DSS in the total cohort, development cohort and verification cohort

First, we divide the total queue equally into two queues, namely the training cohort and the testing cohort. In the training cohort, we sequentially screened for ESRG-associated LncRNA using uni-cox, LASSO (Fig. 3A, B) and mul-cox regression analysis. In the end, we obtained two key LncRNA and constructed a EALncRI based on the results of the mul-cox regression analysis (Supplementary Table 3). The specific formula was followed: EALncRI = expression of MIR4713HG*1.056 + expression of ZEB1-AS1*1.629. In addition, we also analysed the expression correlation of MIR4713HG and ZEB1-AS1 with CERG (Fig. 3C). Before performing prognostic analysis, we first observed the distribution of clinical characteristics between the training cohort and the testing cohort, and we found that there was no difference in the distribution of clinicopathological characteristics between the two (Table 1). Next, we divided the cohort according to the median EALncRI value and performed a prognostic analysis. We found that the high EALncRI group had shorter OS and DSS compared with the low EALncRI group in the total cohort, training cohort, and test cohort (Fig. 3D-I).

**Table 1:** Clinicopathological characteristics of the total cohort, test cohort, and training cohort

Covariates	Type	Total	Test	Training	*p* value
Age	<=65	199(44.42%)	99(44.2%)	100(44.64%)	1
	> 65	249(55.58%)	125(55.8%)	124(55.36%)	
Gender	FEMALE	203(45.31%)	100(44.64%)	103(45.98%)	0.8495
	MALE	245(54.69%)	124(55.36%)	121(54.02%)	
Stage	Stage I	80(17.86%)	41(18.3%)	39(17.41%)	0.2302
	Stage II	166(37.05%)	89(39.73%)	77(34.38%)	
	Stage III	120(26.79%)	61(27.23%)	59(26.34%)	
	Stage IV	67(14.96%)	26(11.61%)	41(18.3%)	
	unknow	15(3.35%)	7(3.12%)	8(3.57%)	
T	T1	14(3.12%)	7(3.12%)	7(3.12%)	0.8358
	T2	81(18.08%)	38(16.96%)	43(19.2%)	
	T3	306(68.3%)	155(69.2%)	151(67.41%)	
	T4	46(10.27%)	24(10.71%)	22(9.82%)	
	Tis	1(0.22%)	0(0%)	1(0.45%)	
M	M0	336(75%)	176(78.57%)	160(71.43%)	0.0971
	M1	66(14.68%)	25(11.16%)	41(18.3%)	
	MX	39 (9.57%)	20(8.93%)	19(8.48%)	
	unknow	7(1.56%)	3(1.34%)	4(1.79%)	
N	N0	262(58.48%)	134(61.61%)	142(55.36%)	0.3406
	N1	109(24.33%)	56(21.43%)	57(27.23%)	
	N2	76(16.96%)	44(16.52%)	36(17.41%)	
	NX	1(0.22%)	1(0.45%)	0(0%)	

### Systematic analysis of EALncRI and clinical parameters

**Fig. 4:**
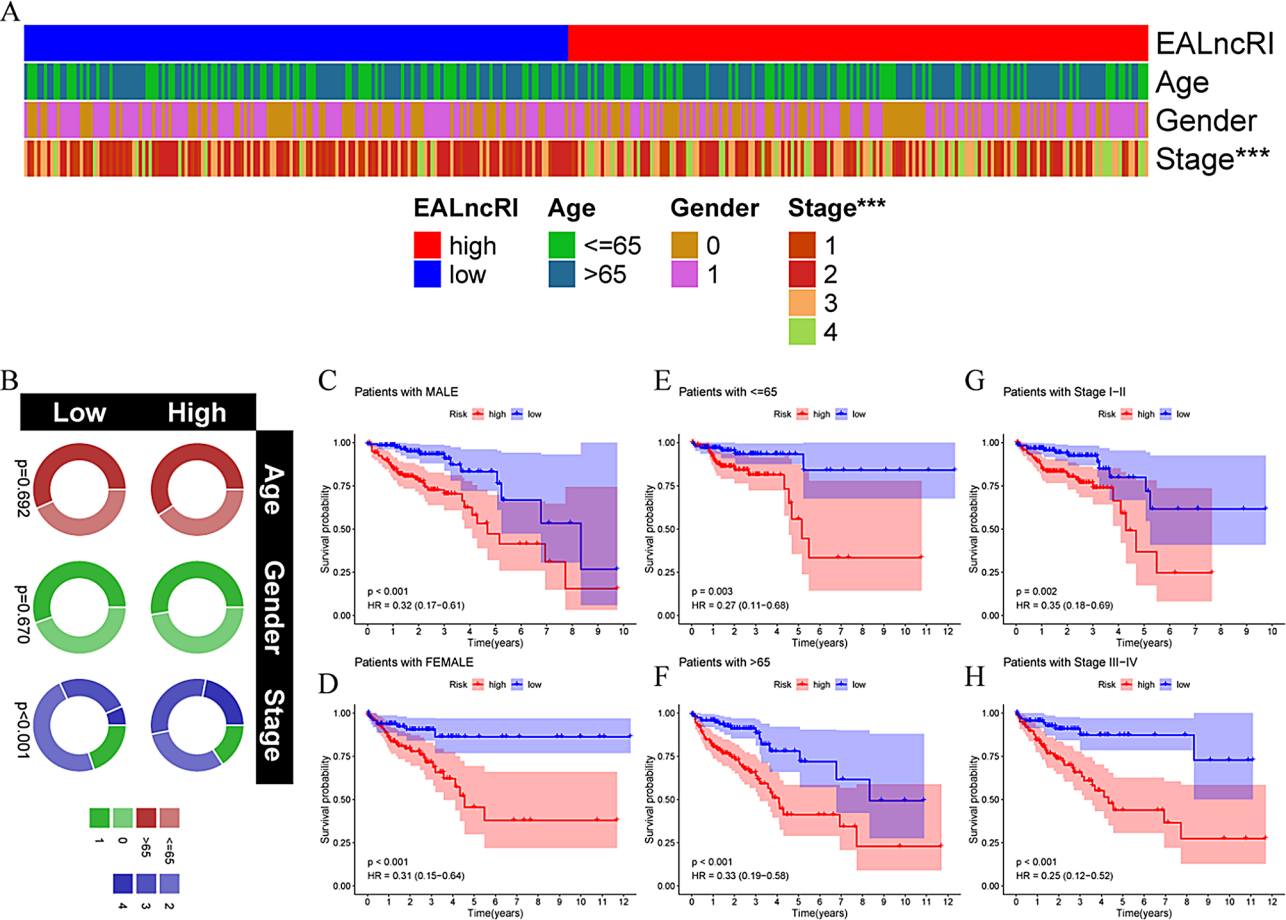
Clinical comprehensive analysis of EALncRI. (**A**-**B**) Heatmap and circle plot of the clinicopathological distribution of the high EALncRI group and the low EALncRI group. (**C**-**D**) KM analysis of the high EALncRI group and low EALncRI group in different sex statuses. (**E**-**F**) KM analysis of the high EALncRI group and low EALncRI group in different age states. (**G**-**H**) KM analysis of the high EALncRI group and low EALncRI group in different tumor stages

First, we visualized the distribution of common clinicopathological features across different EALncRI subgroups using clinical correlation heatmaps and bubble plots. We found that the tumor stages of CRC patients in the low EALncRI group were significantly lower than those in the high EALncRI group (Fig. 4A-B). In addition, we performed subgroup prognostic analysis on CRC patients with different sexes, ages and tumor stages. We found that among CRC patients with different clinicopathological parameters, the prognosis of patients in the low EALncRI group was better than that of patients in the high EALncRI group (Fig. 4C–H). These results indicated that our constructed EALncRI could accurately predict the prognosis with different clinical characteristics.

### Nomogram construction

**Fig. 5:**
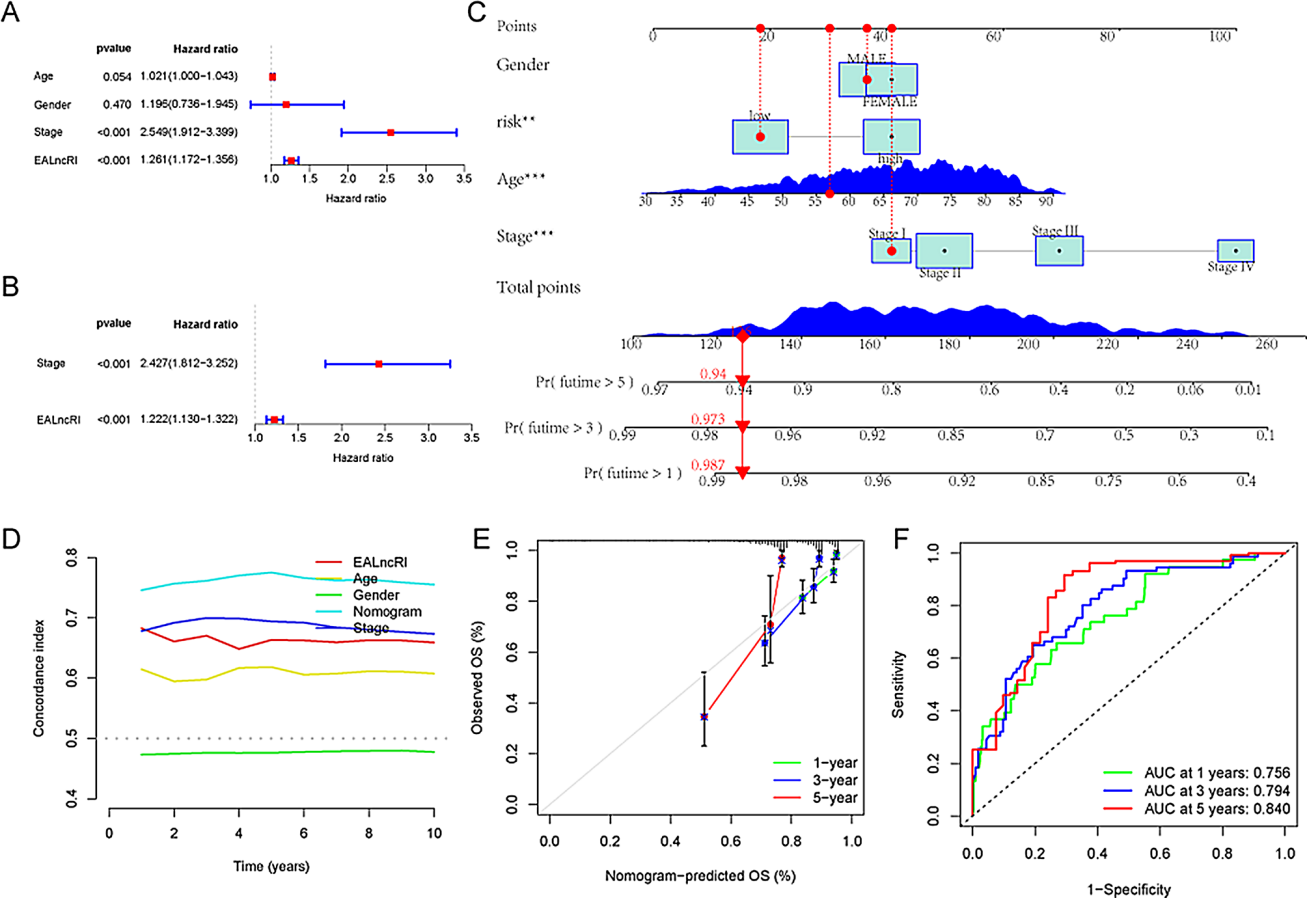
Construction of the nomogram. (**A**-**B**) Univariate and multivariate Cox regression analysis based on MIR4713HG. (**C**) Nomogram combining common clinicopathological parameters and EALncRI. (**D**) c-index of the EALncRI, common clinicopathological parameters, and nomogram. (**E**) Calibration curves when the nomogram predicts 1-year, 3-year, and 5-year OS. (**F**) ROC when the nomogram predicts 1-year, 3-year, and 5-year OS

To further evaluate the prognostic value of EALncRI in CRC patients, we performed univariate and multivariate Cox regression analysis and found that after removing confounding factors such as age, gender, and tumor stage, EALncRI was still an independent risk factor affecting the prognosis of CRC patients. In addition, in order to construct a more stable and accurate prognostic indicator, which is more conducive to clinical application, we combined EALncRI with common clinical pathological parameters (age, gender, and tumor stage) to construct a new nomogram (Fig. 5A). We used the c-index, calibration curve, and ROC to evaluate the accuracy and stability of the nomogram (Fig. 5B-C). We found that the nomogram can predict the prognosis of CRC patients more accurately, effectively, and conveniently.

### GO and KEGG analysis related to EALncRI

**Fig. 6:**
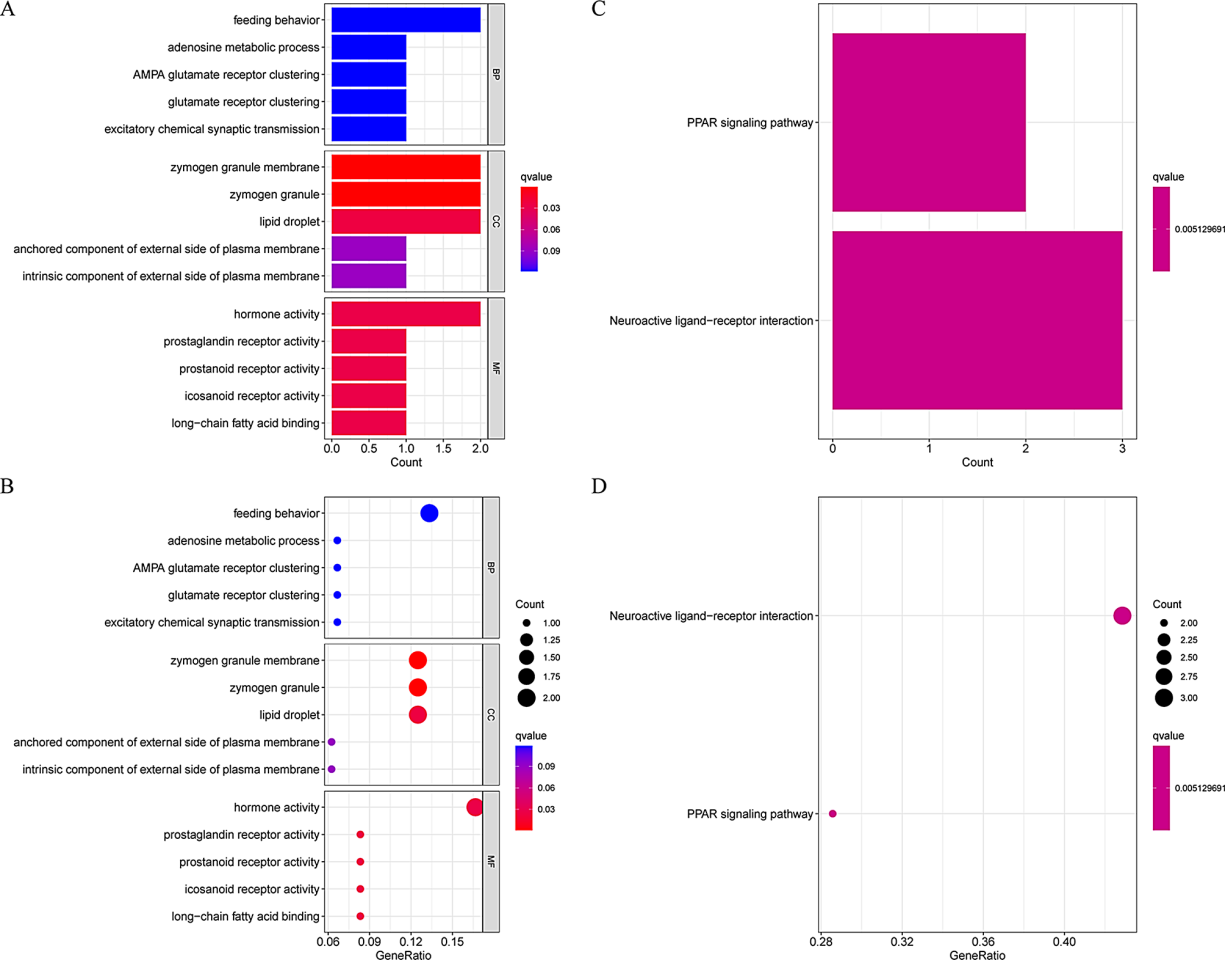
GO analysis and KEGG analysis. (**A**-**B**) Bar and bubble graphs for GO analysis. (**C**-**D**) Bar and bubble graphs for KEGG analysis

In order to explore the biological function differences between the low EALncRI group and the high EALncRI group, we analyzed the DEGs between the two groups and finally obtained 47 DEGs. Based on these DEGs, we performed GO and KEGG analysis. GO analysis showed that, in terms of biological processes only, mainly enriched in and feeding behavior, adenosine metabolic process, AMPA glutamate receptor clustering, glutamate receptor clustering and excitatory chemical synaptic transmission. In terms of cell composition, mainly enriched in zymogen granule membranes, zymogen granules, lipid droplets, anchored components of the external side of the plasma membrane and intrinsic components of the external side of the plasma membrane. In terms of molecular functions, mainly enriched in hormone activity, prostaglandin receptor activity, prostanoid receptor activity, eicosanoid receptor activity and long − chain fatty acid binding (Fig. 6A-B). In addition, KEGG results showed that the main enriched signaling pathways of DEGs were the PPAR signaling pathway and neuroactive ligand − receptor interaction (Fig. 6C-D).

### Comprehensive analysis of EALncRI, the tumor microenvironment

**Fig. 7:**
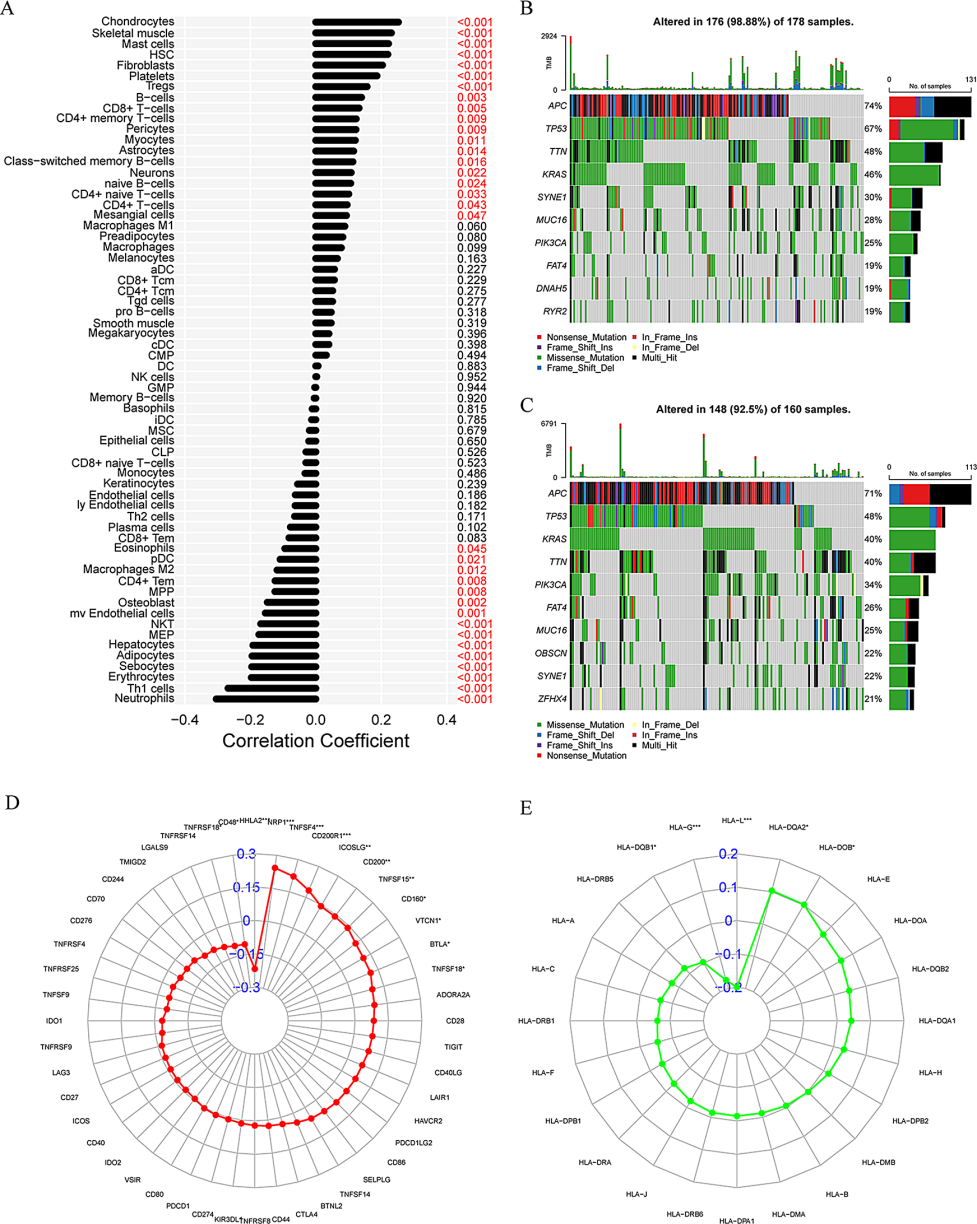
Comprehensive analysis of the tumor microenvironment. (**A**) Correlation analysis between EALncRI and immune cells. (**B**-**C**) Top 10 mutation-prone genes in the high EALncRI group and low EALncRI group. (**D**) Correlation radar map of EALncRI with immune checkpoint molecules. (**E**) Correlation radar map of EALncRI with HLA family gene molecules

First, we evaluated the infiltration levels of immune and stromal cells in CRC tissues using the xCELL algorithm, and explored the correlation between EALncRI and the infiltration levels of immune and stromal cells. The results revealed that EALncRI was negatively correlated with 15 types of cells and positively correlated with 19 types of cells (Fig. 7A). We then explored the genetic mutation profile of CRC and analyzed the 10 genes most susceptible to mutations in the low EALncRI group and the high EALncRI group (Fig. 7B–C). The results showed that in terms of TP53, the mutation rate in the low EALncRI group was significantly lower than that in the high EALncRI group. In addition, I also analyzed the correlation between EALncRI and related molecules (ICM and HLA molecules) that have great guiding significance for immunotherapy. We found that EALncRI was significantly correlated with the expression of 13 ICM and 5 HLA molecules (Fig. 7D-E).

### MIR4713HG used for prognostic evaluation of immunotherapy patients

**Fig. 8:**
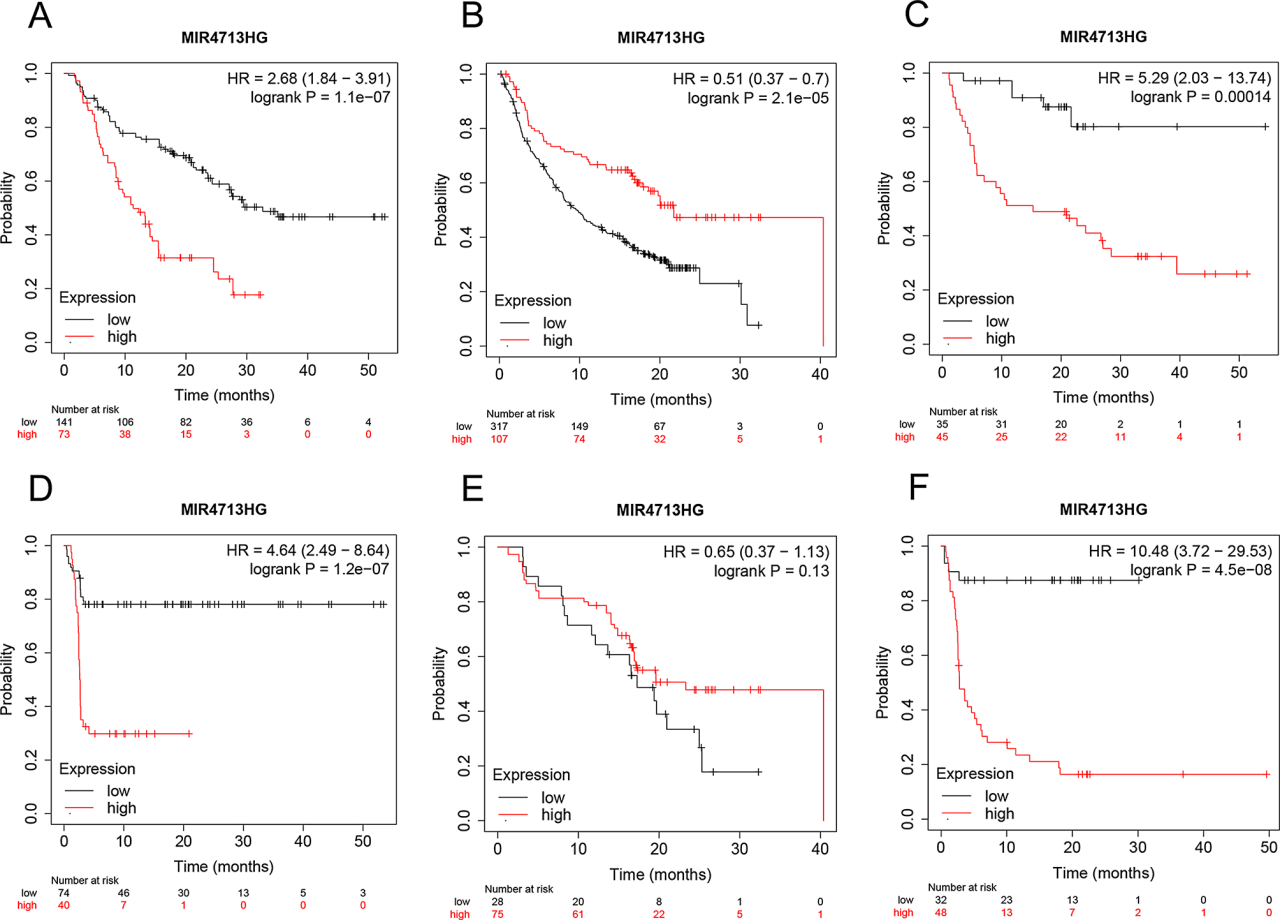
Prognostic value of MIR4713HG in CRC patients receiving immunotherapy. (**A**) MIR4713HG accurately and effectively differentiates OS in patients receiving anti-PD-1-treated patients. (**B**) MIR4713HG accurately and effectively differentiates OS in patients receiving anti-PD-L1-treated patients. (**C**) MIR4713HG accurately and effectively differentiates OS in patients receiving anti-CTLA-4-treated patients. (**D**) MIR4713HG accurately and effectively differentiates PFS in patients receiving anti-PD-1-treated patients. (**E**) MIR4713HG accurately and effectively differentiates PFS in patients receiving anti-PD-L1-treated patients. (**F**) MIR4713HG accurately and effectively differentiates PFS in patients receiving anti-CTLA-4-treated patients

Since MIR4713HG has not been reported in CRC, we focused our research on MIR4713HG. KM-plotter is an excellent and user-friendly online database that provides data from real-world studies evaluating immunotherapy, which included anti-PD-1 treatment patients (*n* = 851), anti-PD-L1 treatment patients (*n* = 486) and anti-CTLA-4 treatment patients (*n* = 131). For immunotherapy evaluation, the expression of MIR4713HG showed good predictive value for OS in patients receiving anti-PD-1 therapy (Fig. 8A), anti-PD-L1 treatment (Fig. 8B), and anti-CTLA-4 treatment (Fig. 8C). Regarding progression-free survival (PFS), MIR4713HG also showed good predictive value for OS in patients receiving anti-PD-1 therapy (Fig. 8D) and anti-CTLA-4 treatment (Fig. 8F). For anti-PD-L1 treatment (Fig. 8E), it was not very good.

### MIR4713HG regulates the oncogenic ability of CRC cells in vitro and in vivo

**Fig. 9:**
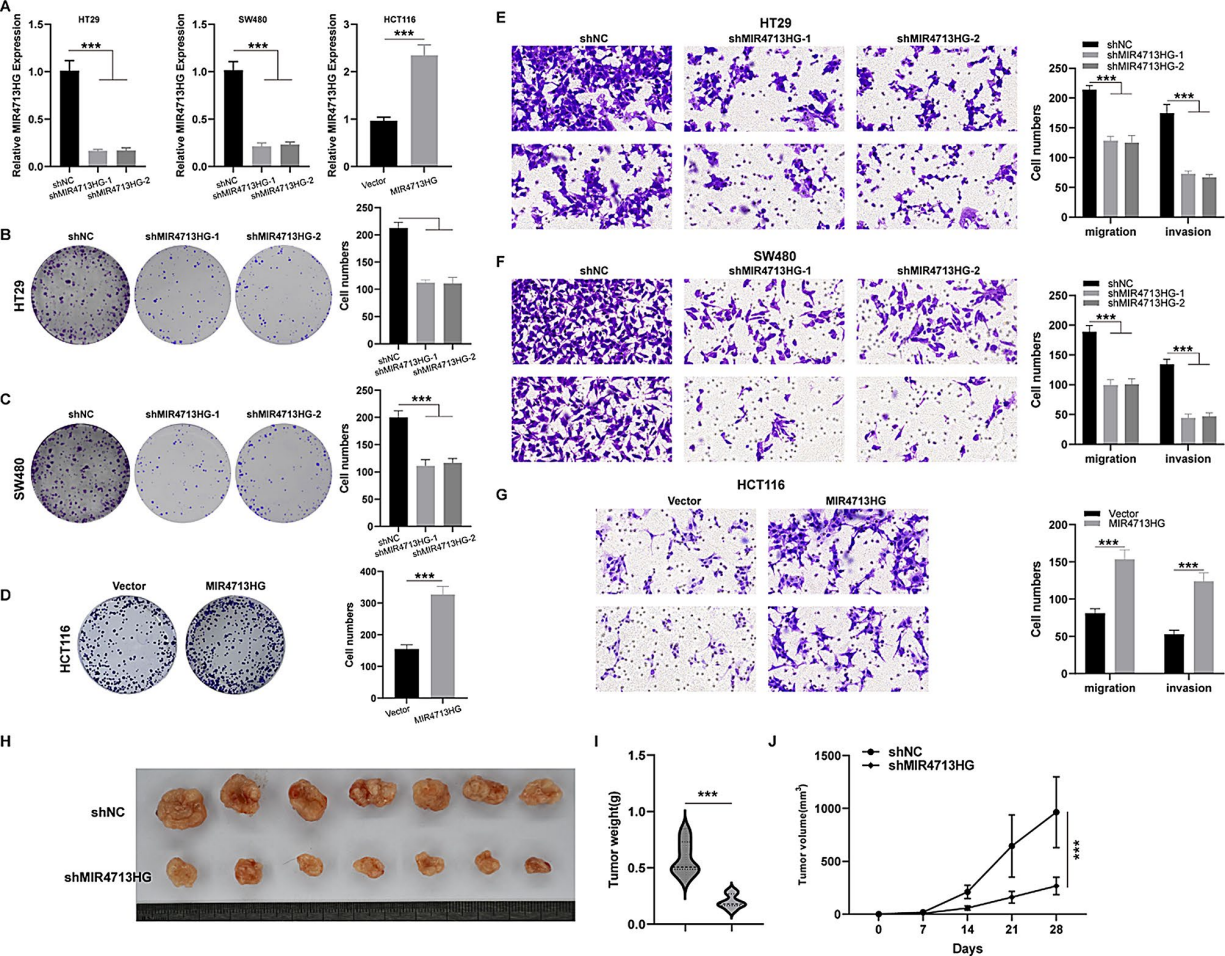
Oncogenic role of MIR4713HG in CRC. (**A**) Efficiency of knockdown and overexpression of MIR4713HG. (**B**-**D**) Effects of knockdown or overexpression of MIR4713HG on the proliferation ability of COAD cells. (**E**-**F**) Effects of knockdown or overexpression of MIR4713HG on the migration and invasion of COAD cells. (**G**-**J**) Effects of knockdown of MIR4713HG on the in vivo growth ability of COAD cells

In addition, we further explored the oncogenic role of MIR4713HG in CRC. We knocked down MIR4713HG expression in HT29 and SW480, and overexpressed MIR4713HG in HCT116, and the transfection efficiency was verified by qPCR (Fig. 9A). Then, we detected the effect of MIR4713HG expression on the proliferation ability of CRC cells by colony formation assay. We found that the cell proliferation ability was significantly reduced after knocking down MIR4713HG (HT29 and SW480), while the cell proliferation ability was significantly enhanced after overexpression of MIR4713HG (HCT116) (Fig. 9B-D). Meanwhile, we detected the effect of MIR4713HG on the migration and invasion ability of CRC cell lines by Transwell assay. We found that the cell migration and invasion ability decreased after knocking down MIR4713HG (HT29 and SW480), and the cell migration and invasion ability was enhanced after overexpression of MIR4713HG (HT29) (Fig. 9E-G). On this basis, we further evaluated the effect of MIR4713HG on tumor growth activity using a xenograft model. The results showed that after MIR4713HG knockdown, the growth ability of subcutaneous tumors in nude mice was significantly impaired (Fig. 9 H-J).

### GO and KEGG analysis related to EALncRI

**Fig. 10:**
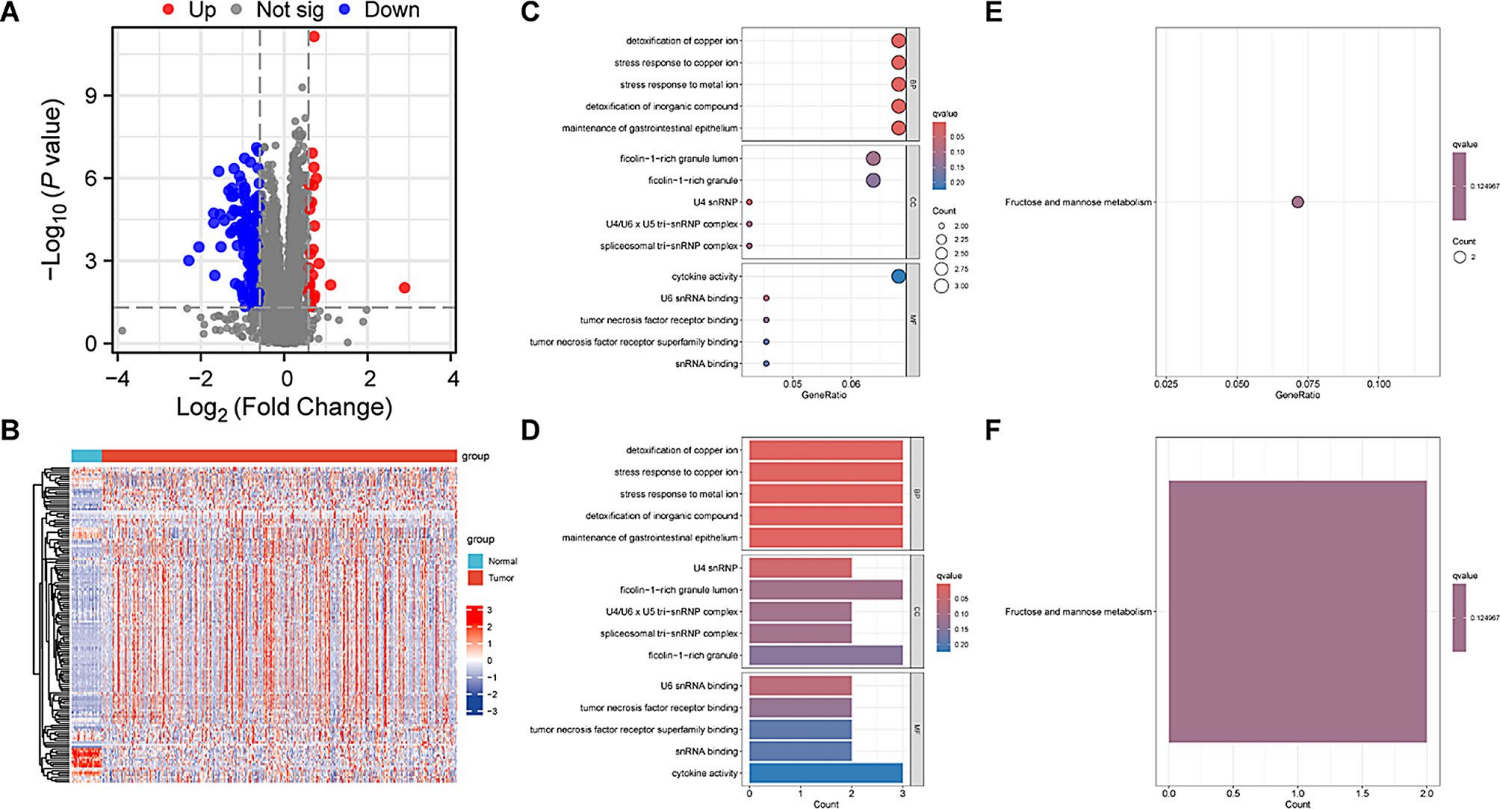
Biological analysis of MIR4713HG. (**A**) Volcano plot of DEGs among different MIR4713HG expression groups. (**B**) Heat map of DEGs expression in TCGA-CRC transcriptome. (**C**-**D**) Bar and bubble plots of GO analysis. (**E**-**F**) Bar and bubble plots of KEGG analysis

In order to explore the biological function of low MIR4713HG, we analyzed the transcriptome data according to the expression of MIR4713HG in TCGA-CRC, and finally obtained 146 DEGs (Fig. 10A). On the one hand, we displayed the expression of 146 DEGs through heat maps (Fig. 10B), and on the other hand, GO and KEGG analysis were performed based on these DEGs. GO analysis showed that in terms of biological processes alone, detoxification of copper ion, stress response to copper ion, stress response to metal ion, detoxification of inorganic compound, maintenance of gastrointestinal epithelium was mainly enriched. In terms of cellular composition, U4 snRNP, ficolin − 1−rich granule lumen, U4/U6 x U5 tri − snRNP complex, spliceosomal tri − snRNP complex, ficolin − 1−rich granule were mainly enriched. In terms of molecular function, U6 snRNA binding, tumor necrosis factor receptor binding, tumor necrosis factor receptor superfamily binding, snRNA binding, cytokine activity was mainly enriched (Fig. 10C-D). In addition, KEGG results showed that the main enriched signaling pathways of DEGs were Fructose and mannose metabolism (Fig. 10E-F).

### Comprehensive analysis of EALncRI, MIR4713HG and drug treatment

**Fig. 11:**
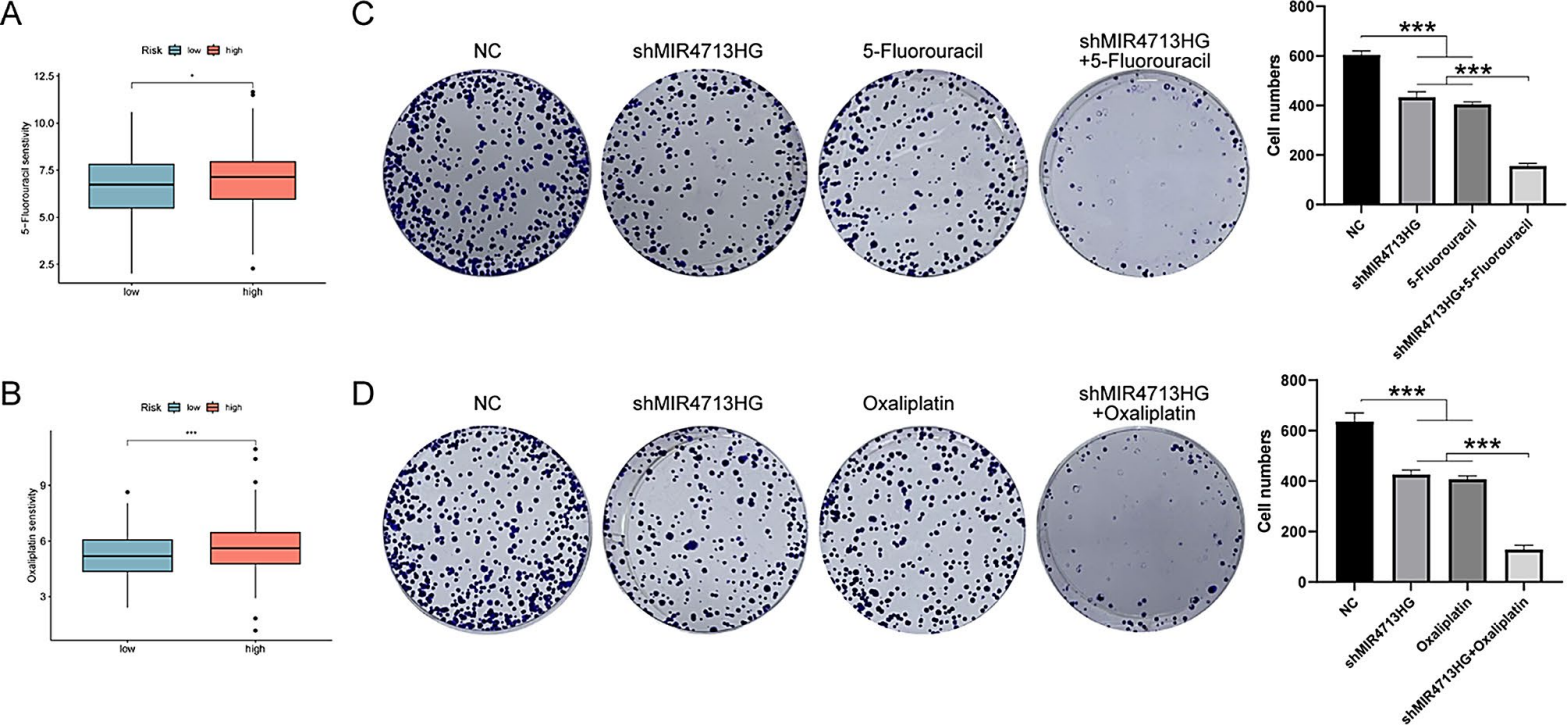
Study on the correlation between EALncRI and drug sensitivity. (**A**-**B**) Differences in sensitivity to 5-fluorouracil and oxaliplatin between CRC patients in the high EALncRI group and the low EALncRI group. (**C**-**D**) Colony formation assay was performed to explore the synergistic effect of knocking down EALncRI and chemotherapy drugs on the proliferation ability of CRC cells

In addition, we analyzed the differences in IC50 values of 2 common CRC chemotherapy drugs (5-fluorouracil and oxaliplatin) between different EALncRI groups. We found that patients in the low EALncRI group may be more sensitive to 5-fluorouracil and oxaliplatin (Fig. 11A-B). At the same time, we found through colony formation assay that knocking down the key gene MIR4713HG in the model has the potential to synergistically inhibit CRC cell tumor growth with 5-fluorouracil or oxaliplatin (Fig. 11C-D).

## Discussion

Using genomic information from the TCGA database to develop biomarkers is a common strategy for predicting cancer prognosis and evaluating the immune microenvironment [25–29]. Many studies currently focus on the role of exosomes and LncRNA in cancer [12, 30, 31]. Therefore, it is necessary to link exosome genes and LncRNA, and it is very important to find exosome-related LncRNA that play a key role. However, systematic studies on exosome-associated LncRNA in CRC are still lacking.

In this research, we performed a comprehensive analysis of 120 ERGs. Through differential analysis, SVM-RFE machine learning, and uni-cox, the key prognostic ERG was screened out. On this basis, we established a key ERG-lncRNA coexpression network. After systematic screening, we finally obtained 2 key LncRNA (MIR4713HG and ZEB1-AS1) and constructed EALncRI. It is worth emphasizing that EALncRI is easier to apply clinically than the prognostic models constructed by other research institutes because it consists of only two LncRNA. CRC patients were divided into low EALncRI group and high EALncRI group according to the median EALncRI value. We found that in the total cohort, test cohort, and training cohort, the low EALncRI group had longer OS and DSS than the high EALncRI group. In addition, under different clinicopathological characteristics, the OS of in the high EALncRI group was significantly shorter than that of the low EALncRI group. Nomograms are widely used to predict the risk and prognosis of various diseases [32–34]. In this study, we constructed a new nomogram based on EALncRI that can accurately predict the prognosis of CRC patients.

We explored potential signaling pathways that EALncRI may affect through DEGs among different EALncRI groups. Our analysis included GO and KEGG analysis, and the results showed that EALncRI may affect the progression of CRC through the PPAR signaling pathway.

At the same time, our study showed that EALncRI can effectively distinguish the tumor microenvironment of CRC, and EALncRI is closely related to the infiltration level of various cells. Furthermore, previous studies have shown that ICM and HLA genes can effectively predict the immunotherapy response [35]. Therefore, we analysed the correlation between EALncRI and immune response predictive markers. The results showed that EALncRI was closely related to the expression of multiple ICM and HLA genes. These results imply that EALncRI may provide partial insights for guiding immunotherapy in CRC patients. More importantly, the study results that the incidence of TP53 mutation in the low EALncRI group was significantly lower than that in the high EALncRI group. In addition, studies have shown that TP53 is the most commonly mutated gene in CRC and often signifies a poor prognosis [36]. This may be one of the factors leading to the poor prognosis of patients in the high EALncRI group.

5-Fluorouracil and oxaliplatin are classic drugs for the clinical treatment of CRC [37, 38]. Therefore, we calculated the drug sensitivity to 5-fluorouracil and oxaliplatin in CRC patients in different EALncRI groups. The results suggest that CRC patients in the low EALncRI group are more suitable for 5-fluorouracil and oxaliplatin than the high EALncRI group. These findings prevent the occurrence of clinical drug resistance.

More importantly, we found that the model gene ZEB1-AS1 has been studied in CRC [39], while MIR4713HG has not been reported in CRC. Therefore, we conducted an in-depth study on MIR4713HG in CRC. Our results showed that knockdown of MIR4713HG expression inhibited the proliferation, migration and invasion abilities of the CRC cell SW620 and HCT116. In addition, reduced expression of MIR4713HG can significantly inhibit the tumor growth ability of CRC. In addition, previous studies have shown that MIR4713HG can be loaded by exosomes as “cargo” [40], highlighting its potential application value as a liquid biopsy tool in the diagnosis and treatment of CRC. Based on these results, it can be speculated that MIR4713HG is a promising therapeutic target for CRC. Previous studies suggest [41–44] that that RNA nanotherapeutics targeting MIR4713HG may provide a new theoretical basis for clinical decision-making for CRC patients in the future.

Similar to previous studies, this study also has some shortcomings. First, the risk score EALncRI developed in this study was only validated in an internal cohort, not in an external cohort. In addition, the carcinogenic mechanism of MIR4713HG in CRC still needs to be further verified in the future.

To sum up, our study constructed a prognostic marker EALncRI that can accurately predict the prognosis of CRC. On this basis, an easy-to-apply nomogram was further constructed. This study also explored the correlation between EALncRI and the tumor microenvironment, providing some guidance for immunotherapy and chemotherapy in CRC patients. Finally, we performed *in vitro and in vivo* experiments to confirm the carcinogenesis of MIR4713HG in CRC.

## Electronic supplementary material

Below is the link to the electronic supplementary material.

Supplementary Material 1

Supplementary Material 2

Supplementary Material 3

## Data Availability

No datasets were generated or analysed during the current study.

## Abbreviations

CRC: Colorectal cancer
lncRNA: Long noncoding RNA
EALncRI: lncRNA risk index
ERGs: Exosome-related genes
scRNA-seq: Single-cell RNA sequencing
CERG: Core ERG
Uni-cox: Univariate Cox
Mul-cox: Multivariate Cox
EALncRI: Exosome-associated lncRNA risk index
DEGs: Differentially expressed genes
ICM: Immune checkpoint molecules
KM: Kaplan-Meier
